# Programmes of Societies

**Published:** 1984-10

**Authors:** 


### Programmes of Societies, 1984–85

### BRISTOL MEDICO-CHIRURGICAL SOCIETY

Meetings
1984–1985

1984

Wednesday 10th October
Annual General Meeting and President's Address

Wednesday 14th November
Long Fox Lecture. Professor I. A. Silver 'Animals and Medicine'

Wednesday 12th December
Clinical Meeting at Manor Park Hospital

1985

Wednesday 9th January
Mr. K. D. J. Vowles – 'Surgical Problems in the Aged'
*Southmead Hospital*

Wednesday 13th February
Professor G. K. Wilcock, Professor in Care of the Elderly – 'Care of the Elderly – the way ahead?'
*Frenchay Hospital*

Wednesday 13th March
Dr. E. D. Acheson, Chief Medical Officer, D.H.S.S. – 'Hospitals – Past, Present and Future'
*Medical School*

Wednesday 10th April
Mrs. H. Eason – 'Bristol's Historic Inns'
*Southmead Hospital*

Wednesday 8th May
Visit to the new Jenner Museum, The Chantry, Berkeley

### BRITISH MEDICAL ASSOCIATION
### BRISTOL DIVISION

Meetings
1984–1985

1984

Sunday 28th October. 11 a.m.
St. Luke's Tide Service
*Cathedral Church of St. Peter and St. Paul, Clifton*

Friday 9th November
President's Reception and Dinner
Principal Guest – Professor Kenneth Rawnsley (recently President of Royal College of Psychiatrists)
*Brentry Hospital*

Friday 7th December
A Wine Tasting –
arranged by Messrs Hicks & Don
*Southmead Centre of Medical Education*

Thursday 31st January
'Jesus – The Evidence'
Ian Wilson, author of 'The Turin Shroud'.
*Southmead Centre of Medical Education*

Thursday 21st February
Dr. Keable-Elliott will speak
Treasurer of B.M.A.
*Southmead Centre of Medical Education*

Friday 29th March
'Surgical Experiences in India'
Mr. Christopher Gordon Russell, Consulting Surgeon, Middlesex Hospital
*Southmead Centre of Medical Education*

Thursday 25th April
Mr. L. W. Curson
Manager of B.U.P.A.
*Southmead Centre of Medical Education*

Wednesday 5th June
Summer Reception
*Dyrham Park*

Wednesday 24th October
Annual General Meeting
*Southmead Centre of Medical Education*

[May 1985 – a further meeting may be arranged].

